# Anatomical factors in medication-related osteonecrosis of the jaws

**DOI:** 10.1080/07853890.2021.1897445

**Published:** 2021-09-28

**Authors:** J. A. Correia, A. Lóio, I. A. Furtado, A. Capelo, C. Caldas, N. Santos, F. Salvado

**Affiliations:** a University Clinic of Stomatology, Faculty of Medicine, University of Lisbon, Lisbon, Portugal; b Stomatology Department, Centro Hospitalar Universitário Lisboa Norte, Lisbon, Portugal; c Institute of Anatomy, Faculty of Medicine, University of Lisbon, Lisbon, Portugal; Department of Medicine; d Faculty of Life Sciences, University of Madeira, Funchal, Portugal; e Centro de Investigação Interdisciplinar Egas Moniz (CiiEM), Egas Moniz Cooperativa de Ensino Superior, Caparica, Portugal

## Abstract

**Introduction:**

Medication-related osteonecrosis of the jaws (MRONJ) is a serious adverse event of antiresorptive and antiangiogenic drugs that affects bone and soft tissue of the maxilofacial region [1]. Clinical features and risk factors of MRONJ have been described in several publications, however few studies address the anatomical factors involved [2,3]. This study aims to: (a) describe in detail the anatomical location of MRONJ lesions; (b) identify the most susceptible areas and association with other factors.

**Materials and methods:**

A retrospective study was conducted including all patients with MRONJ diagnosis in an Oral Surgery Clinic between 2004 and 2018. The data was collected from the patient clinical records. Lesion extension was determined by the physical exam and/or computer tomography. Statistical significance was defined as *p* < .05. Chi-square, Student *t*-test, ANOVA and log-linear analysis were used as appropriate. All were performed using IBM SPSS® version 23.

**Results:**

A total of 147 patients were included in the sample, 95 (64.6%) females and 52 (35.4%) males, with a mean age at diagnosis of 68.12 ± 11.02 years. A total of 182 lesions were diagnosed, 67 (36.8%) in the maxilla and 115 (63.2%) in the mandible. The molars and premolars regions were most affected both in the maxilla (1st and 3rd sextants) with 46 (68.7%) lesions, and in the mandible (4th and 6th sextants) with 96 lesions (83.4%). The alveolar bone was affected in 170 (93.9%) the lesions, whereas 18 (9.9%) had involvement of the basal bone, 7 (3.8%) arose in the mylohyoid line and only 5 (2.7%) occurred in the hard palate. At least one of the cortical walls was involved in 125 (68.7%) lesions, the buccal wall in 53 (29.1%) lesions, the palatal or lingual wall in 32 (17.6%) lesions and both walls in 40 (22%) lesions. Male patients were more likely to have multiple lesions located both in the maxilla and mandible (17.3%) than female patients (5.3%) (*p* < .017). In absence of any traumatic factor, periodontal disease was found in 34.4% of patients with anterior lesion location (2nd or 5th sextants) vs 10.2% with posterior lesion location, although not statistically significant (*p* < .89). We found no statistically significant differences or association between lesion location or lesion extension and dentoalveolar surgery, use of dental prosthesis, age at diagnosis, type of drug and length of antiresorptive or antiangiogenic medication.

**Discussion and conclusions:**

MRONJ lesions were more frequently located in the mandible as expected from previous studies [2,3]. The molars and premolars regions were most affected and at least one cortical wall was involved in most lesions. Some authors have concluded that areas with thin mucosa are more susceptible, our results are in accordance as we identified several lesions in the mylohyoid line [3]. Our study results can reflect the importance of anatomical factors such as occlusal forces, blood supply and bone density vary in these locations.
